# The subjective metric of remembered colors: A Fisher-information analysis of the geometry of human chromatic memory

**DOI:** 10.1371/journal.pone.0207992

**Published:** 2019-01-02

**Authors:** María da Fonseca, Nicolás Vattuone, Federico Clavero, Rodrigo Echeveste, Inés Samengo

**Affiliations:** 1 Departamento de Física Médica, Centro Atómico Bariloche e Instituto Balseiro, Comisión Nacional de Energía Atómica, Universidad Nacional de Cuyo, Bariloche, Argentina; 2 Consejo Nacional de Investigaciones Científicas y Técnicas, Bariloche, Argentina; Universitat de Valencia, SPAIN

## Abstract

In order to explore the metric structure of the space of remembered colors, a computer game was designed, where players with normal color vision had to store a color in memory, and later retrieve it by selecting the best match out of a continuum of alternatives. All tested subjects exhibited evidence of focal colors in their mnemonic strategy. We found no concluding evidence that the focal colors of different players tended to cluster around universal prototypes. Based on the Fisher metric, for each subject we defined a notion of distance in color space that captured the accuracy with which similar colors where discriminated or confounded when stored and retrieved from memory. The notions of distance obtained for different players were remarkably similar. Finally, for each player, we constructed a new color scale, in which colors are memorized and retrieved with uniform accuracy.

## 1 Introduction

There is a long-standing controversy regarding the computational strategies employed by humans to process color. In one classical research line [1–5], the accuracy with which similar colors are discriminated was explored using purely perceptual tasks that do not require additional cognitive processing, such as language or memory. Other approaches, in contrast, specifically explored how the continuum of hues was partitioned into discrete categories, often corresponding to the linguistic labels of different colors. Some studies concluded that the segmentation process does not depend on linguistic labels, [6–8], whereas others argued that linguistic labels do indeed give rise to different chromatic category boundaries [9–13, 13–15], sometimes even differentiating between representations in the low (sensory discrimination) or the high (attention, language) level of processing [16]. Still other studies report mixed results that show the effect in some (but not all) category boundaries [17], that may depend on the degree of training [18]. The physiology of human ventral V4 even provides evidence that the representation of color changes, depending on whether the task does or does not involve linguistic components [19].

The different results obtained by different studies [20] have lead to postulate the hypothesis that a given color-processing experiment may or may not reveal evidence of chromatic categorization depending on the cognitive computations required by the task [10, 21, 22]. In this view, the presence of categorical boundaries in one particular experiment does not imply that the same boundaries must be found in other experiments [23]. This hypothesis challenges the assumption that color is represented with a unique neural code, and rather suggests that there may be several simultaneous—and not necessarily equivalent—representational drafts [24]. Some of these drafts may become instrumental in language mediated tasks, and remain dormant in purely perceptual tasks, and vice versa. The codes that operate in different tasks should hence be analyzed separately. In this study we set out to investigate the strategies that require the transient storage and retrieval of color from memory, since it is not clear whether chromatic memory is organized in terms of a few focal colors, or rather makes use of the continuum of possible hues. In several previous studies [6, 9, 11, 12, 15, 25–27], the strategy was to assess the accuracy of chromatic memory throughout color space. Typically, uneven results were obtained, with some regions of color space inducing accurate retrieval, but not others. The goal was then to determine whether such regional variations could be related to the color categories induced by language, i. e., whether retrieval error was larger at the focal color of each category, and smaller at the boundary between two categories, even if the task itself was not cued with linguistic labels. Taking a more neutral approach to the possible origin of categories, here we defined categories in terms of response properties, not linguistic labels. We designed a computer game in which players had to store a color in memory. After a short period of time viewing a distractor screen, players were asked to retrieve the stored color among a continuum of possibilities. The task per se did not force players to use any linguistic-based segmentation of colors. Participants, however, were free to employ the mnemonic strategy of their preference, which could, in principle, be based on a language-based tactic, or on associations with specific well-remembered objects that could act as a reference.

On one extreme, a completely unstructured mnemonic strategy is possible, by which the player memorizes and retrieves an unbiased representation of each color. In this case, the retrieval errors corresponding to a fix target color have zero mean, and a variance that represents the necessarily limited accuracy of the storage and retrieval process. If the variance is constant throughout color space, the mnemonic accuracy of the player is uniform. If the variance varies from color to color, the strategy is not completely unstructured, since the chromatic memory of the player is capable of making finer discriminations in some regions of color space than in others.

On the opposite extreme, a completely categorical strategy is possible, by which the player divides the continuum of hues into discrete categories, and only memorizes the category. When asked to retrieve the original color, the player may use different procedures to select the responded color from the remembered category. They may use a representative color for each category, also called a *focal color*, and use this color as the referent of the whole category. Alternatively, they may retrieve a color chosen randomly within the category. For a fixed target color, the probability distribution of the response may or may not be flat. A flat distribution has no focal colors, but still retains category boundaries. A delta-shaped distribution in which all the probability is concentrated at certain specific colors is both categorical and organized in terms of attractors, or foci. All these categorical strategies, however, produce retrieval errors that do not average out to zero, at least, for all the stored colors that do not coincide with a focal color. The uncovering of non-zero average retrieval errors, hence, is a symptom of a mnemonic strategy based on the existence of categories. Therefore, in this paper, we carefully assess whether mean retrieval errors are significantly different from zero. If they are, this condition is taken as a symptom of a categorical strategy.

Among the previous studies that addressed the effect of categories in chromatic memory, the most relevant for the present paper are two analyses of Bae et al [26, 27], since they tested retrieval accuracy using a continuous repertoire of colors forming a closed locus that contained all hues. Their main finding is that the accuracy of chromatic memory is not uniform throughout color space. Both the mean and the variance of retrieval errors vary systematically with the color stored in memory. The variations are consistent throughout the sample, and they are related to purely perceptual variations. Here, we expand those initiatives in three new directions. First, we test color memory in a closed locus in color space, in which equi-distant steps are defined by an objective criterion. Second, we assess individual differences between observers. Third, chromatic memory is explored with the specific aim of constructing, for each observer, a mnemonically uniform chromatic scale.

The natural tool to construct this scale is the so-called *Fisher information* [28]. This tool can be used to bound the maximal accuracy with which two remembered colors can be discriminated [29]. An additional—and less advertised—functionality of the Fisher information, is that it constitutes a metric, allowing us to calculate distances between pairs of colors [30, 31]. As opposed to, for example, the Euclidean distance, lengths based on the Fisher metric represent how differently each individual observer holds the two colors, in terms of his or her ability to discriminate them in mnemonic tasks. Moreover, individually tailored notions of distance can be used to build a new, mnemonically uniform color scale for each observer. This scale may be useful both from a theoretical and a practical perspective. From the theoretical persepctive, assessing individual differences in the mnemonically uniform chromatic scale of different observers may provide information about the generality of the mechanisms underlying the representation of colors in memory. From the practical perspective, individually tailored mnemonically uniform scales could be useful, for example, to design applications for cell phones and computer screens that define the colors of stimuli intended to be memorized in such a way that any observer (including color blind people) can maximally profit from mnemonically discrimination abilities.

In this paper, we use the experimental data gathered from a color memory task to characterize the strategies used by humans to store and recall colors. We find evidence that individual subjects employ category-based strategies, and we calculate the Fisher metric for each observer. Although we find no conclusive evidence that different players build categories around universal focal colors, we do observe some regularities in the mnemonic discrimination ability of the tested subjects. These regularities give rise to individually tailored mnemonically uniform color scales that are fairly similar across the sample of tested observers.

## 2 Results

### 2.1 A computer game to test chromatic memory

**Fig 1:**
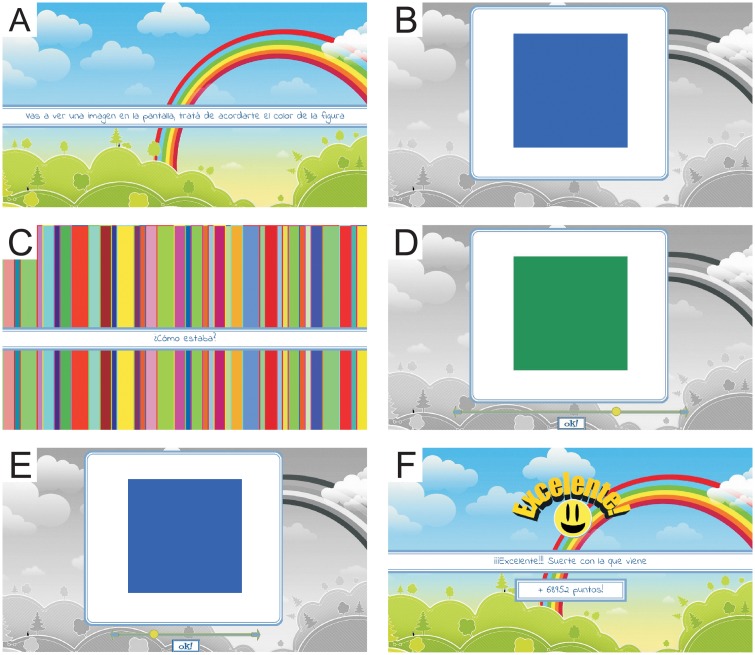
Organization of each memory test. A: Explanatory screen where the player is instructed to remember the color shown next. B: Target color to be remembered. C: Mask containing the instruction to recover the target color in the screen shown next. D and E: Searching screens, where the player continuously changes the color of the central square by displacing the cursor in the bottom bar. The aim is to recover the target color shown before (panel B). The selected color is chosen by clicking on the button labeled “Ok!”. F: Feedback screen, where the expression on the emoticon (that may vary from happy to sad) and the obtained punctuation are determined by the similarity between the target and the responded colors.

The computer game consisted of a sequence of memory tests. Players sat at approximately 60 cm from the computer screen. Fig 1 illustrates the structure of each test. First, playerd were instructed to remember the color that would be shown next, the so-called *target* color.

Immediately after, the screen displayed an 11 cm × 11 cm square filled with the target color for 2.5 sec (Fig 1B). The area of the square was chosen large and the background achromatic, so as to minimize perceptual shifts due to contrasts [32, 33]. Then, a random Mondrian screen lasting for 5 sec separated the target color from the test period (Fig 1C). The 5-second interval of the mask ensured that retrieval happened once memories were stabilized [34], and the Mondrian display was intended to eliminate the afterimages introduced by uniformly colored masks [35]. In the test period, a second colored square appeared (Fig 1D), the hue of which varied continuously, as the player displaced a cursor on a bar. The bar swept over 743 possible responded colors. The task was to move the cursor until the target color was recovered. The player scrolled freely along the bar for as long as they wished (typically a few seconds), until he or she identified the color considered to be the best match to the target color. The choice was reported by clicking on an *Ok!* button (Fig 1E). The score of the trial depended on the distance between the target color and the responded color. The player received feedback on their performance in the form of numerical points, and also with an emoticon whose expression depended on the score (Fig 1F). Feedback was included because it helped players maintain their attention in an otherwise boring game, and its presence probably also improved performance [36]. Each game tested 32 target colors, the order of which was randomly selected at the beginning of each game. Each subject played the full 32-target game at least 10 times.

### 2.2 The choice of color coordinates

Experiments aiming at revealing categorical effects in color perception, color discrimination or color memorization must always be reported in specific color coordinates. The choice of coordinates has a profound impact on the reported accuracy of performance, since accuracy is typically informed with some measure of distance in color space. When the color coordinates are changed with a nonlinear transformation, expanding certain regions of color space and contracting others, distances change non-uniformly. These alterations only depend on the transformation, and not on the way information is processed by the visual system. There is previous evidence [26, 34] that results vary when reported in different color coordinates. Response distributions that appear broad in one set of coordinates become narrow in another, and vice versa. When these discrepancies are consistent across subjects, it is difficult to conclude whether the shape of the measured distributions reveals a property of the visual system of all observers, or a property of the chosen coordinates. When only a single set of coordinates is considered, but multiple behavioral tasks are tested [17, 27], distributions that are narrow (or broad) consistently across tasks may indicate that all tasks share the same metric properties, or alternatively, that the coordinates where colors are represented allocate a small (large) volume to a region where the visual system is precise (imprecise).

To avoid the arbitrariness of the choice of coordinates, many studies report the analysis of higher cognitive functions (as color naming, color categorization, or chromatic memory) using a scale of colors where chromatic perception is supposedly uniform, as the OSA System, or CIELUV, or CIELAB. The alleged uniformity, however, is only approximate [17, 26, 37]. Another possibility is to work in the DKL space, constructed to represent the natural scale of geniculate neurons [38]. Perception, however, is not uniform in this scale either, since discrimination ellipses vary in size and eccentricity from point to point [32, 39]. Moreover, in the DKL space, the iso-luminant plane is only approximately L + M, and the degree of involvement of cones S is observer-dependent [40]. In fact, there is ample evidence that even beyond luminance, and irrespective of the chosen coordinates, there are significant perceptual differences among trichromats [26, 39, 41–44], implying that there is no single coordinate system that can be viewed as perceptually uniform by several observers. One way to tackle this problem is to define color coordinates individually tailored for each observer [31]. This solution, however, does not allow results to be reported in an objective set of coordinates.

For these reasons, in the present study, and following previous policies [26], we opt to be cautious, and not to emphasize the size of retrieval accuracy in different regions of color space. Our main objective, however, is to construct a mnemonically uniform set of coordinates for each observer, and to assess the significance of the differences between observers. These aims can be reached irrespective of the initial color coordinates (see below), so perceptual uniformity is not mandatory in our study. In view of the aforementioned shortcomings of the allegedly perceptually uniform color coordinates, and of the main focus of the present study, we selected the color locus of the experiment using physical criteria to fixate the total brightness, the set of hues, and the scale along the curve. Coordinates were chosen with the following requirements:

1. All colors of the locus of tested target and response colors had the same light intensity, measured as the amount of energy within the visible range.
2. In order to work with the widest possible collection of hues available in our computer monitor, all colors on the locus were maximally saturated, so that at least one of the coordinates *R*, *G* or *B* vanished.
3. Equally-spaced steps along the locus corresponded to shifting a fixed amount of energy from one range of wavelengths to another. That is, two consecutive colors on the locus were chosen in such a way that the integral of the modulus of the difference of their spectra remained constant.

Physical criteria 1-3 do not produce colors that are perceptually uniform, so they have the drawback of not allowing a neat separation between perception and memory. The advantage is that there is an objective logic behind the resulting metric. The procedure required to construct the chromatic locus is described in Sect. 4.1.

The set of target colors *t* tested in the experiment formed a closed locus in color space. All colors on the locus had equal light intensity and maximal saturation, and the distance between two neighboring colors was proportional to the difference between the two spectral densities (see Methods, Sec. 4.1). The colors on a closed locus can always be parametrized with a phase *t* ∈ (− *π*, *π*]. We arbitrarily assign *t* = 0 to a deep shade of blue, and as a consequence, the opposite color *t* = ±*π* corresponds to red. For each target color *t*, a player generated *n* responses, with *n* ranging between 10 and 26 (mean 12, SD 5.2). The sample average $\bar{r}\left(t\right)$ of these responses was calculated for each player and each *t*, together with the sample variance *ϵ*^2^(*t*).

### 2.3 Characterizing the players’ responses

**Fig 2:**
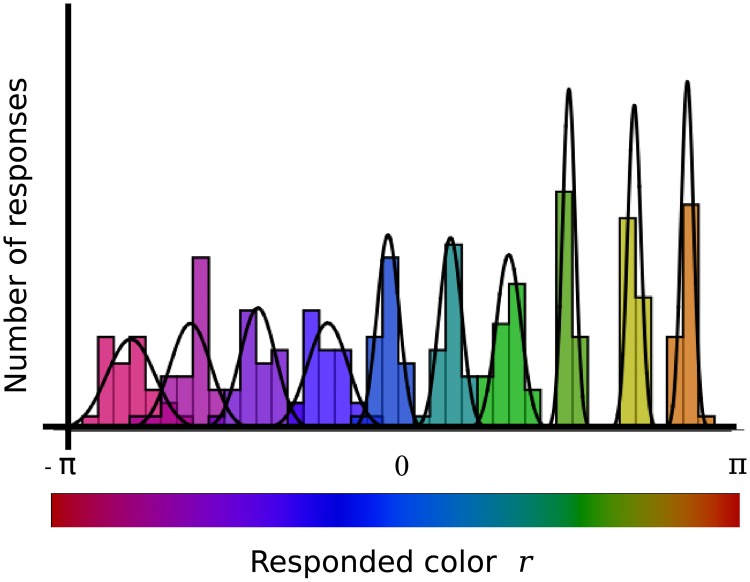
Histograms of responses conditional to a fixed target color. A subset of 10 histograms (out of the tested 32, one per target color) obtained when sampling the probability *P*(*r*|*t*) of responding color *r* when presented with target *t*. Each curve is obtained for a different *t*, for a subject who played the game 26 times.

The computer game was played by 11 subjects (see Sect. 4.2). The probability of retrieving color *r* when the target color was *t* is *P*(*r*|*t*). A perfect player has *P*(*r*|*t*) = *δ*(*r* − *t*). Inaccuracies in the retrieval process are due to the fact that *P*(*r*|*t*) differs from *δ*(*r* − *t*). In Fig 2 the response histograms of an example player are displayed.

The unimodal nature of the histograms implies they can be well approximated by a Gaussian function

$$\begin{array}{c} P\left(r|t\right)=\frac{e^{-{\left[r-\mu \left(t\right)\right]}^{2}/2\sigma^{2}\left(t\right)}}{\sqrt{2\pi \sigma^{2}\left(t\right)}} \end{array}$$ (1)

of mean *μ*(*t*) and variance *σ*^2^(*t*). All other players yielded qualitatively similar results.

Inaccuracies in retrieval can be attributed to two different causes:

- *μ*(*t*) may differ from *t*, implying there is a bias: When color *t* is stored in memory, the retrieved color *r* is on average shifted from *t*.
- *σ*(*t*)>0, so there is trial-to-trial variability in the retrieved colors.

The first cause gives rise to systematic errors, whereas the second, to fluctuations.

**Fig 3:**
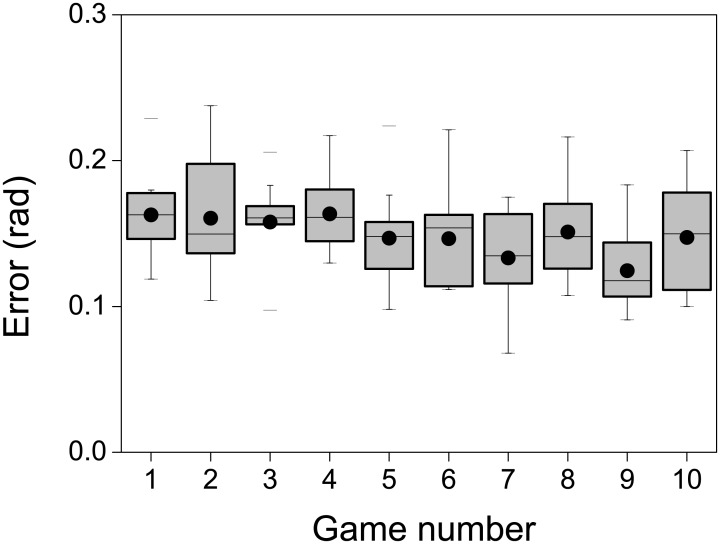
Constancy of the response error throughout the playing history. Box histograms of the sample distribution of the absolute value of the error, averaged in target colors, in 10 consecutive runs of the game. The error of player *i* in the *k*-th game is defined as $\langle |r_{k}^{i}\left(j\right)-t\left(j\right)|\rangle$, where the angular brackets represent an average over the 32 target colors *j*. Each box represents the histogram collected from the 11 players (*i* ∈ [1, 11]). Dot: sample mean. Horizontal line: sample median. Upper and lower borders of the box: 25% and 75% percentile of the responses. Whiskers: 5% and 95%. Horizontal bars: maximum and minimum responses.

Averaging in target colors and in subjects, the absolute value of the difference between the responded color and the target color was approximately 0.15 radians (≈ 9°), an amount that is of the order of the angular separation of two consecutive target colors (2*π*/32 ≈ 11°). The subject-to-subject variability of the absolute value of the error (measured as the standard deviation) was 0.023 radians (≈ 1.3°), implying that the tested sample of players displayed a rather uniform response accuracy. Since the maximum error was *π*, the mean error was ∼ 5% of the maximum attainable. No evidence was found of improving performance with practice, as can be deduced from Fig 3. We conclude that players constructed their strategy in the very first trials, with no relevant modification thereafter. The essentially flat behavior of the boxes of Fig 3 implies that the steady state was reached rapidly.

Hence, there is no need to discard the initial responses due to a transient learning process. In the rest of the paper, we focus on the dependence of response errors on the target color.

Averaging in target colors and in subjects, the mean standard deviation of the responses was 0.17 radians (≈ 9.7°). A Gaussian function with a standard deviation of 0.17 radians contains 99% of its mass in a region of angular width 0.88 radians, which represents the 14% of the interval (− *π*, *π*]. For all players and all tested colors, hence, the response probabilities *P*(*r*|*t*) were concentrated around the mean, and the tails were not wide enough to notice the circular nature of the variable *r*. The maximal standard deviation (the subject and color with widest distribution) was 0.85 radians (≈ 48°), implying that the widest distribution concentrates 99% of its mass around 27% of the available interval (−*π*, *π*]. These response accuracies justify the choice of non circular functions, as Gaussians, to fit the response probabilities. Gaussians are defined on the set (−∞, +∞), whereas responded colors belong to the locus (−*π*, *π*]. Hence, circular distributions, as for example von Mises’, are in principle better suited for the angular variable *r*. Yet, the small value the standard deviations implies that for all practical purposes, the Gaussian approximations coincided with a von Mises distribution of the same mean and variance. We chose to work with the Gaussian approximation, since it is analytically tractable (see Sect. 2.5). A Smirnov-Kolmogorov check for gaussianity of the 352 recorded distributions (32 per player) showed that all data sets were compatible with the Gaussian hypothesis (*p*-value of 0.05, corrected for multiple comparisons).

**Fig 4:**
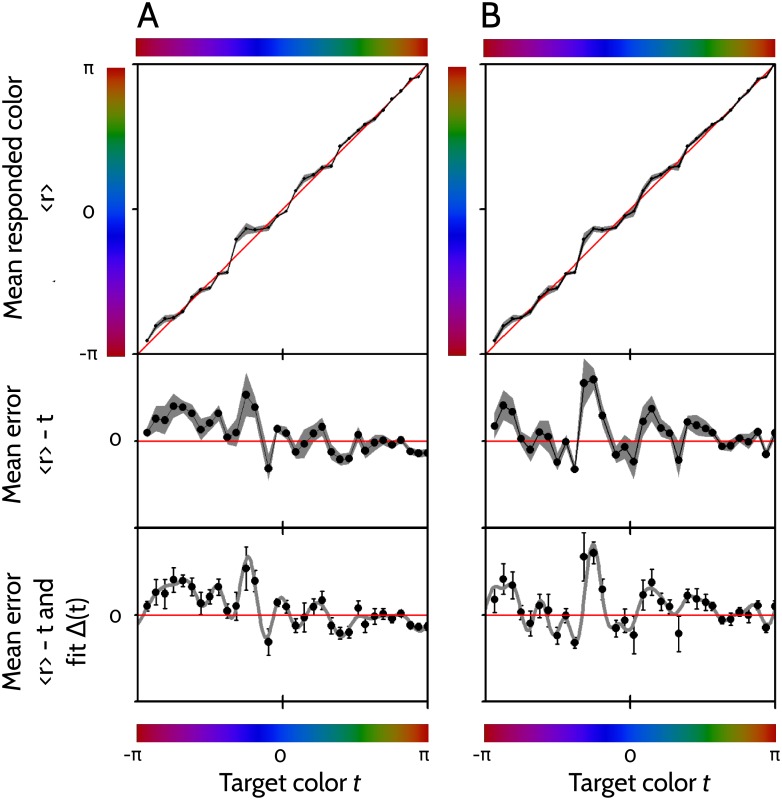
Characterization of the responses. Panels A and B correspond to two example players. Top: Black line: Mean response $\bar{r}$ as a function of the target color *t*. Gray area: Range of values in $\bar{r}\left(t\right)\pm \varepsilon \left(t\right)/\sqrt{n}$. Middle: Mean error $\bar{r}\left(t\right)-t$ (black line), together with expected error of the mean $\varepsilon \left(t\right)/\sqrt{n}$ (gray area). Bottom: Fitted curve Δ(*t*), together with the experimental data $\bar{r}\left(t\right)-t$ (black dots) and the expected error of the mean $\varepsilon \left(t\right)/\sqrt{n}$ (error bars). In the middle and bottom panels, the vertical scale spans from −*π*/6 to + *π*/6.

At the top panels of Fig 4, the mean responses $\bar{r}\left(t\right)$ of two example players are displayed, together with the expected error of the mean $\varepsilon \left(t\right)/\sqrt{n}$, where *n* is the number of times the game was played.

For some colors, the departure of $\bar{r}\left(t\right)\pm \varepsilon \left(t\right)/\sqrt{n}$ from the target color *t* suggests that the responded color deviates systematically from the target. To confirm whether such deviations are significant, and to evaluate whether this effect is also verified in other players, for each subject we evaluated the null hypothesis that the mean responses $\bar{r}\left(t\right)$ were equal to the test colors *t* with added Gaussian noise $\varepsilon \left(t\right)/\sqrt{n(t)}$. Performing a chi-squared test on the accumulated squared error

$$\begin{array}{c} S=\sum_{i=1}^{32}{\left[\frac{\bar{r}\left(t\right)-t}{\epsilon \left(t\right)/\sqrt{n(t)}}\right]}^{2}, \end{array}$$ (2)

using a *p*_*value*_ of 0.01, and then correcting it to account for Bonferoni’s multiple tests, we rejected the null hypothesis for all subjects. In other words, the discrepancy between $\bar{r}\left(t\right)$ and *t* was significantly greater than expected by fluctuations of the order of the standard deviation $\varepsilon \left(t\right)/\sqrt{n(t)}$ alone. We conclude that it would be remarkably unlikely to obtain such systematic errors from a limited number of samples of a Gaussian function centered in *t*.

**Fig 5:**
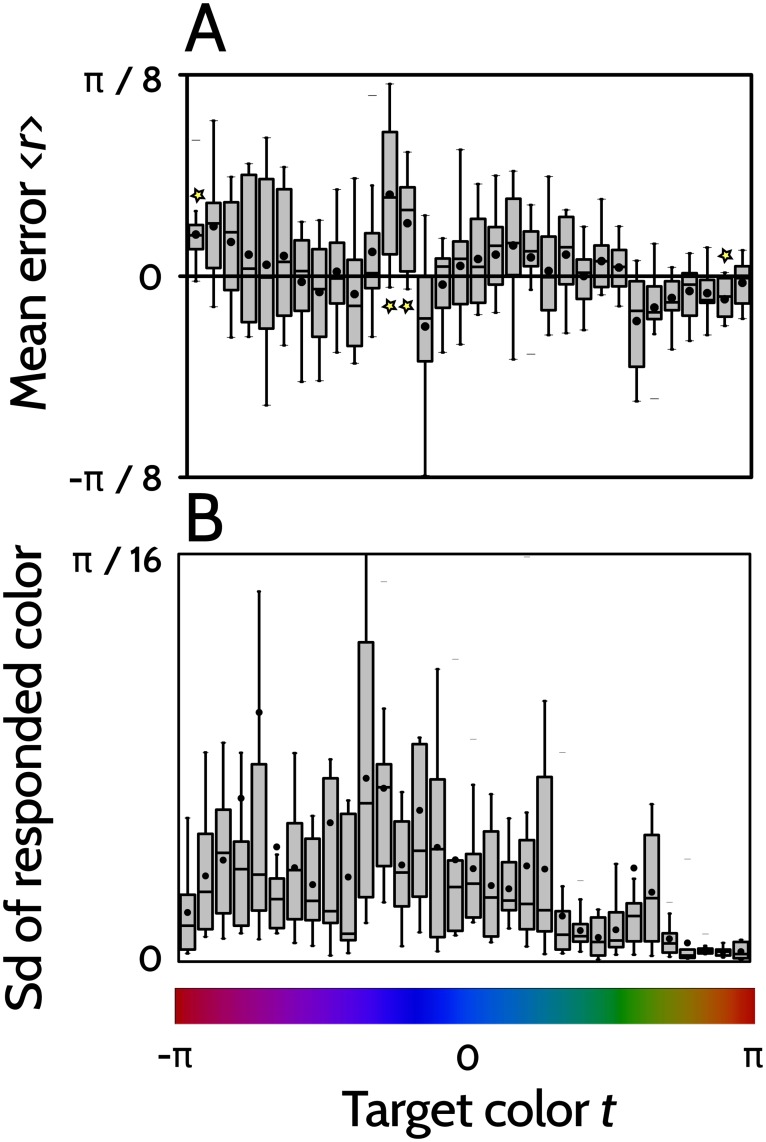
Sample statistics of the recorded responses. Sample histogram of the mean error $\bar{r}\left(t\right)-t$ (panel A), and of the responses’ standard deviation *ϵ*(*t*) (panel B) for the 11 players. In A, the yellow stars indicate the target colors for which a two-sided *t*-test evaluating whether $\bar{r}-t$ is significantly different from zero yields a particularly small *p*_value_. From left to right: *p* = 0.004, 0.004, 0.004 and 0.002. Boxed-histogram conventions same as in Fig 4.

Both example players make positive errors around the blue-violet border. Moreover, for both of them the standard deviation seems to become particularly small in the yellow-orange zone. In order to assess whether these characteristics also held with other players, we calculated the sample histograms of the mean response error $\bar{r}\left(t\right)-t$ and of the standard deviation of responses *ϵ*(*t*) as a function of the target color *t*, as shown in Fig 5.

Panel A confirms that for the collection of sampled payers, specific colors are associated with specific errors, for example, in the red-purple and in the violet-blue region, errors tend to be positive, whereas in the orange zone, they tend to be negative (stars in Fig 5A). From panel B we deduce that for the sampled players, the standard deviation is particularly high in the violet-blue region, and particularly low in the yellow-orange zone.

### 2.4 Attractor and repulsor colors

Several studies [6, 9, 11, 12, 15, 25–27] have assessed whether the accuracy of chromatic memory could be related to color categories, the latter defined in terms of linguistic labels. Either the borders of such categories and/or the focal color (or best representative) of each category were determined in one experiment. Then a second experiment was performed, in which the accuracy of chromatic memory was evaluated (a) on colors that were near a boundary between categories, (b) on colors that were far from a boundary, (c) on colors that were near a focal color, and/or (d) on colors that were far from a focal color. If the accuracy of chromatic memory was confirmed to be modulated by the proximity to category boundaries or to focal colors, the performance of memory was concluded to be influenced by the linguistic segmentation of the continuum of colors.

This paradigm requires two experiments: One to determine category boundaries, or alternatively, category focal colors, and another to measure the accuracy of chromatic memory. The accuracy was assessed using some measure of spread of the colors retrieved in response to a single memorized target, larger spread implying poorer performance. In the present study, and following [26, 27], we changed the classical paradigm in two ways. First, we did not anchor the concept of categories to linguistic labels. This policy was adopted in order to also detect non-linguistic categories. Second, the presence of categories was not assessed by changes in the spread of the colors retrieved in response to a single target, but by changes in their mean. As explained below, certain systematic variations in the mean allow us to define the concept of attractor colors and of repulsor colors for each player, both of which can be linked to the concept of categorical memory. The advantage of assessing categorization with a measure defined in terms of the mean (as opposed to the spread) is that the mean is less sensitive to the choice of coordinates than the spread. Imagine, for example, that when memorizing a certain shade of blue, a given player tends to retrieve colors that are shifted towards the violet side. The spread of the responses may be large or small, depending on the chosen coordinates. In fact, by performing a nonlinear change of coordinates that expands or contracts the responded colors, the spread will appear to increase or diminish accordingly. However, the fact that the majority of responses are systematically biased towards the violet side (as opposed to the green side) does not depend on the choice of coordinates.

If players use a purely categorical strategy to memorize colors, then each target color *t* is represented internally as a member of a category *c*(*t*). If only the category of the color is stored in memory, the mean *μ*(*t*) of the conditional response probability of Eq 1 depends on the target color *t* only through its category, that is, *μ*(*t*) = *μ*[*c*(*t*)]. Under these circumstances, the measured $\bar{r}\left(t\right)$, as displayed in the upper panels of Fig 4, appears as a staircase, with a flat mean response $\bar{r}\left(t\right)$ inside each category *c*(*t*), and a discontinuous jump when passing from one category to the next. The mean error $\bar{r}\left(t\right)-t$, as displayed in the middle panels of Fig 4, appears as a jigsaw, composed of segments of straight lines inside each category, each of them crossing the horizontal axis with slope −1. The point where each segment of the error $\bar{r}\left(t\right)-t$ vanishes can be thought of the center of the corresponding category, because at that point, the mean responded color coincides with the target color. Moreover, the colors where the mean response (and also the mean error) displays an upward discontinuity constitute the borders between categories.

The mean response *μ*(*t*) is only expected to be flat inside each category if players only memorize the category of the target color. This is a rather radical strategy. A more plausible mechanism is that players employ a mixed strategy, so that the mean response *μ*(*t*) is partly determined by the category, and partly by the particular color *t* [27]. In this case, the well defined staircase of the purely categorical case is expected to smooth out up to a certain degree, becoming a continuous function of *t*. Inside each category $\bar{r}\left(t\right)$ will no longer be flat. Yet, the categorical component of the strategy should still be visible in a mean response $\bar{r}\left(t\right)$ that increases slowly with *t*, with a slope that is somewhere between 0 (fully categorical strategy) and 1 (no categories at all). In turn, when crossing a category boundary, $\bar{r}\left(t\right)$ is no longer expected to be discontinuous, but it should still grow with a slope that is larger than unity. These characteristics can also be recognized in the mean response error $\bar{r}\left(t\right)-t$ as segments where the error diminishes and crosses the horizontal axis with a negative slope (inside each category), and segments where the horizontal axis is crossed with a positive slope (transition between categories). These ideas are now formalized by defining attractors and repulsors.

An attractor is a target color *t*_*a*_ that tends to concentrate the responses to neighboring target colors. Mathematically, an attraction implies that target colors lying to the right of *t*_*a*_ elicit responses with a negative mean error, and target colors to the left of *t*_*a*_ elicit responses with a positive mean error. Exactly at *t*_*a*_, the mean error vanishes. The basin of attraction of each attractor corresponds to a color category.

A repulsor color *t*_*r*_, in contrast, tends to defocus responses. In other words, responses to target colors that are close to a repulsor are systematically deviated *away* from the repulsor *t*_*r*_. Mathematically, a repulsion implies that target colors lying to the right of *t*_*a*_ elicit responses with a positive mean error, and target colors to the left of *t*_*a*_ elicit a negative mean error. Again, at *t*_*a*_, the mean error vanishes.

The sign of errors does not depend on the choice of coordinates. If the scale used to measure colors around the locus is changed with an invertible and nonlinear transformation, the magnitude of errors may vary from point to point, but positive errors will remain positive, and negative errors, negative. Hence, the significance with which attractors or repulsors can be detected may suffer, but not their existence, nor their position.

To verify the number and location of attractors and repulsors of a given player, we modeled the mean responses $\bar{r}\left(t\right)$ measured experimentally as a continuous function of *t*

$$\begin{array}{c} \mu (t)=t+\Delta (t), \end{array}$$ (3)

where Δ(*t*) is the difference between the mean response and the target color, and is a periodic function of *t*. As such, it can be modeled as a trigonometric sum

$$\begin{array}{c} \Delta \left(t\right)=\sum_{j=1}^{m}a_{j}\;\text{sin}\left(jt\right)+\sum_{j=0}^{m}b_{j}\;\text{cos}\left(jt\right). \end{array}$$ (4)

In Eq 4, all sums are restricted to fall in the interval (−*π*, *π*]. The number *n* of terms in the sum, as well as the coefficients *a*_*j*_ and *b*_*j*_, are fitted through a Maximum Likelihood procedure described in Methods, Sec. 4.3. The procedure calculates not only the optimal *n*, *a*_*i*_ and *b*_*i*_ values, but also their uncertainties. In the bottom panels of Fig 4 we see the maximum likelihood fit of the errors of the two displayed example players.

For one given player, a target color *t*_a_ was considered to be a candidate attractor if

1 the optimal fit of the error vanished, that is, Δ^opt^(*t*_a_) = 0, and
2 the optimal fit of the error crossed zero with a negative slope, that is, $d\Delta^{\text{opt}}{\left(t\right)/dt|}_{t=t_{a}}<0$.

Repulsors *t*_r_ were defined analogously, but replacing negative derivatives by positive ones.

Caution is required, however, since the fitted model Δ^opt^(*t*) contains a certain level of uncertainty. Even in the hypothetical case that responses were generated from a Gaussian function with mean *μ*(*t*) = *t*, implying that the mean error Δ(*t*) is identically zero, just from the unavoidable fluctuations that appear in any finite sample of responses, the *fitted* Δ(*t*) will most likely *not* vanish, and conditions 1 and 2 will still detect spurious attractors and repulsors. To decide whether a candidate attractor or repulsor is spurious or not, a criterion is needed, ensuring that the negative (or positive) derivative remains negative (positive) when the uncertainties in the fit of Δ(*t*) are taken into account. The coefficients that define the expansion of Δ(*t*) as a trigonometric series have a varying degree of reliability, depending on the size of the error bars of the recorded responses. Maximum likelihood estimation of the coefficients provides not only the value of the optimal coefficients, but also, their degree of reliability. Changing the coefficients of the expansion from their optimal values to some other nearby suboptimal—but still highly probable—values is likely to cause spurious attractors to disappear, but not significant ones. Hence, we add a third condition that screens all attractor (and repulsor) candidates, and only retains as significant those that pass the following criterion:

3 Deviations from the maximum likelihood fitted model Δ(*t*) with the highest 95% of probability also fulfilled condition 2 (see Methods, Sect. 4.4).

We have verified that criteria 1-3 detect no significant attractors and repulsors with simulated subjects whose responses are generated with Gaussian distributions of mean *μ*(*t*) = *t*, playing the same number of games as the real subjects.

**Fig 6:**
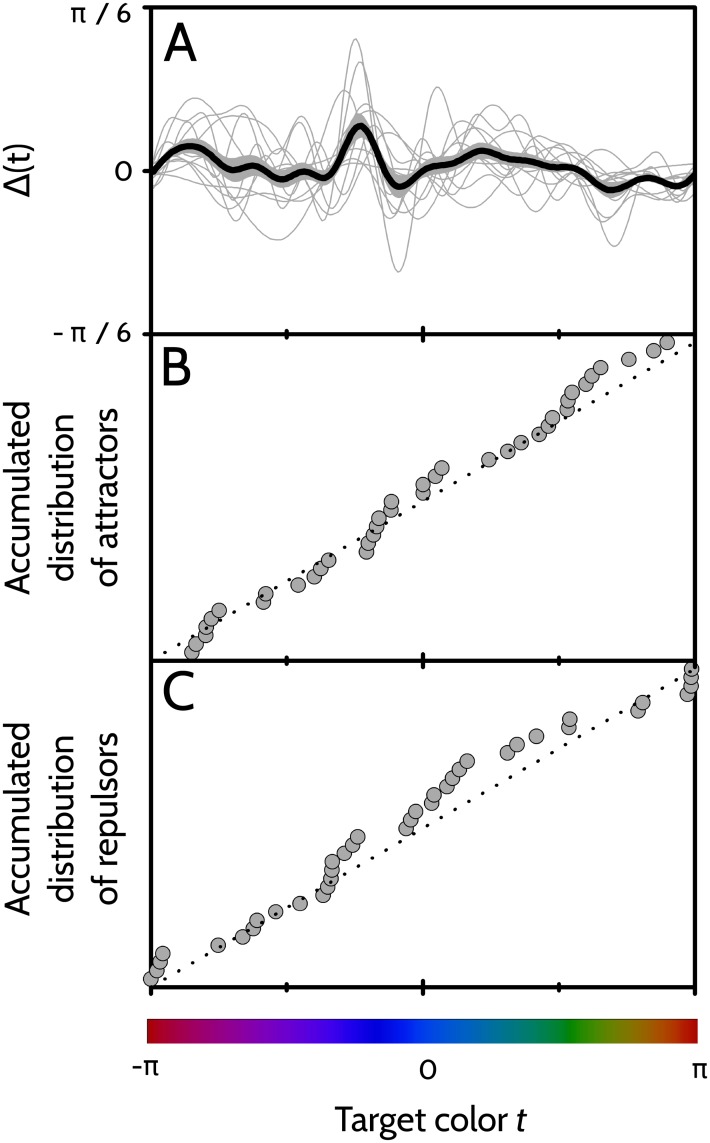
Analysis of attractors and repulsors for the collection of sampled players. A: Thick line: Sample average of the fitted error 〈Δ(*t*)〉. Gray area: Region of values contained in 〈Δ(*t*)〉 ± standard error of the sample mean. Thin lines: individual fits Δ(*t*) for each of the 11 players. B: Data points: Empirical cumulative distribution function of the set of attractors obtained from the 11 players. Dotted line: Accumulated distribution expected for a uniformly distributed process. C: Same as B, for repulsors. In B and C, the vertical axis extends from 0 to 1.

Both the number of attractors per player and the number of repulsors per player ranged between 1 and 6 (mean number of attractors 3.5, SD 1.4, mean number of repulsors 3.5, SD 1.6). In Fig 6A we see the fit Δ(*t*) for all 11 players, and also the sample average.

Players tend to recall attractors, and not to recall repulsors. The set of attractors and repulsors of a player, hence, characterizes his or her personal mnemonic strategy. To assess whether individual strategies are shared by the collection of sampled players, we analyze the empirical accumulated distributions (Fig 6B and 6C) of the attractors and the repulsors of the whole sample. If the set of all attractors and repulsors are scattered uniformly in the interval (−*π*, *π*], there is no universal strategy. In contrast, if attractors and repulsors tend to cluster around specific colors, the hypothesis that human trichromats tend to share the same prototypes (the attractors), and category boundaries (the repulsors) gains strength. In Fig 6B and 6C we compare the empirical cumulative distribution function (gray data points) with the expected accumulated distribution under the uniform hypothesis (dotted line). Clusters, if present, are evidenced as sequences of data points with a significantly larger slope than the dotted line. The Smirnov-Kolmogorov test, however, does not confirm a significant deviation of the data points from the dotted line (*p* = 0.8 for attractors, and *p* = 0.5 for repulsors). Hence, there is no evidence to conclude that individual mnemonic strategies be shared by the sample of 11 subjects.

### 2.5 The metric of remembered colors

The experiment aims at revealing whether chromatic memory is equally accurate throughout color space. In other words, whether a certain color (for example, blue) can be recalled with more or less accuracy than some other color (for example, orange). The magnitude of a mistake is quantified by the distance between the target and the recalled color. This quantification procedure requires a notion of distance in color space. The colors of the experiment were chosen in such a way that when moving from one color to the next, the corresponding spectra were equal, except for a fixed amount of energy that was displaced from the blue LED to the red, or from the red to the green, or from the green to the blue (see Methods, Sec. 4.1). The metric of the game, hence, is defined in terms of physical properties. The recall accuracy of human players, however, need not be constant in this physical metric. Indeed, both the mean and the variance of the experimental data vary with *t*. If the recall accuracy of a given player varies from color to color, one can argue that, from the subjective point of view, the colors of the game are not equi-distant. Those that are recalled accurately can be said to be subjectively distant from their neighbors, whereas those that give rise to errors are subjectively near to their neighbors.

Here we introduce a notion of proximity based on the idea that two colors *t*_1_ and *t*_2_ are to be considered near if the corresponding responses are governed by similar probability distributions *P*(*r*|*t*_1_) ≈ *P*(*r*|*t*_2_). The Fisher information *J*(*t*) is a metric tensor that captures this notion locally. It is defined as the rate of change of the Kullback-Leibler divergence when passing from the probability distribution *P*(*r*|*t*) to the distribution *P*(*r*|*t* + d*t*), that is, (Amari 2000)

$$\begin{array}{c} J\left(t\right)=-\langle \frac{\partial^{2}}{\partial t^{2}}\text{ln}P\left(r|t\right)\rangle , \end{array}$$ (5)

where the mean value is calculated with respect to *P*(*r*|*t*). If the response probability can be modeled by the Gaussian function of Eq 1, the Fisher information becomes

$$\begin{array}{c} J\left(t\right)={\left(\frac{\mu^{\prime }\left(t\right)}{\sigma (t)}\right)}^{2}+\frac{1}{2}{\left(\frac{{\left[\sigma^{2}\left(t\right)\right]}^{\prime }}{\sigma^{2}\left(t\right)}\right)}^{2}. \end{array}$$ (6)

An important property of the Fisher metric is the Crámer-Rao bound, stating that 1/*J*(*t*) is the minimum mean square error that any estimator can make of the target color *t* from the response *r*. In other words, if the Fisher information *J*(*t*) is small for a certain *t*, then the probability *P*(*r*|*t*) hardly varies with *t*, implying it is impossible to make accurate estimates of *t* from *r*. The conclusion holds irrespectively of the decoding algorithm [29, 45]. This property allows us to associate *J*(*t*) with a measure of discriminability. Broadly speaking, *J* is somehow similar to the parameter *κ* controlling the width of Von Mises response distributions in [26], or the inverse of the just-noticeable differences in discrimination experiments in [17]. Yet, from Eq 6, one should bear in mind that variations in *J*(*t*) not only reflect variations in the response variance *σ*^2^(*t*), but also, in the mean response *μ*(*t*).

With the Fisher metric, the distance element between two neighboring colors *t* and *t* + d*t* is

$$\begin{array}{c} ds=\sqrt{J(t)}\;dt, \end{array}$$ (7)

so that the total distance between two target colors *t*_1_ and *t*_2_ is

$$\begin{array}{c} D\left(t_{1},t_{2}\right)=\int_{t_{1}}^{t_{2}}ds=\int_{t_{1}}^{t_{2}}\sqrt{J(t)}\;dt. \end{array}$$ (8)

Replacing Eq 6 into Eq 8,

$$\begin{array}{c} D\left(t_{1},t_{2}\right)=\int_{t_{1}}^{t_{2}}\sqrt{{\left(\frac{\mu^{\prime }\left(t\right)}{\sigma (t)}\right)}^{2}+\frac{1}{2}{\left(\frac{{\left[\sigma^{2}\left(t\right)\right]}^{\prime }}{\sigma^{2}\left(t\right)}\right)}^{2}}\;dt. \end{array}$$ (9)

From Eq 6, we see that the calculation of *J*(*t*) requires the derivatives d*μ*/d*t* and d*σ*^2^/d*t*. Those derivatives were obtained from the analytic fit *μ*(*t*) = *t* + Δ(*t*) of the experimental mean $\bar{r}\left(t\right)$, and another similar fit of the experimental variance *ϵ*^2^(*t*), also modeled as a trigonometric series *σ*^2^(*t*), using the same procedure employed to fit Δ(*t*).

**Fig 7:**
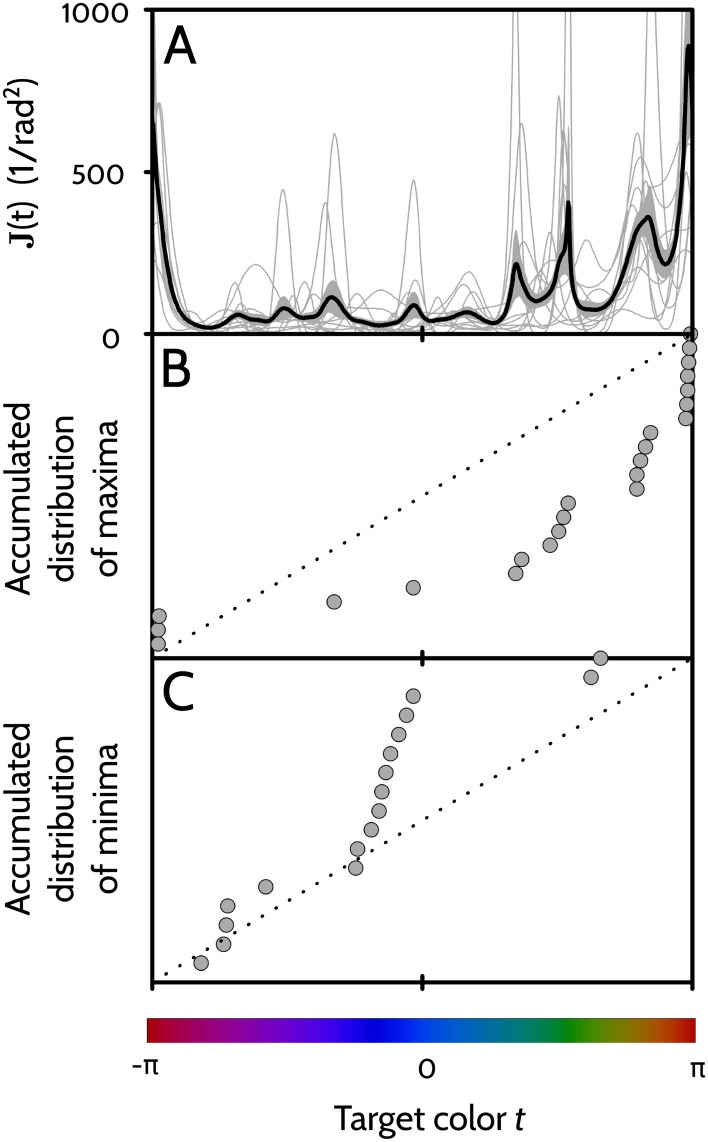
Amount of Fisher information along the locus of sampled colors. A: Thick line: Sample average of the amount of Fisher information obtained by the individual players. Gray area: Region of values contained in 〈*J*(*t*)〉 ± standard error of the sample mean. Thin lines: individual curves *J*(*t*) for each of the 11 players. B: Data points: Empirical cumulative distribution function of the set of relevant maxima of *J*(*t*) obtained from the 11 players. Dotted line: Accumulated distribution expected for a process with uniform probability density. C: Data points: Empirical cumulative distribution function of the set of relevant maxima of 1/*J*(*t*) obtained from the 11 players. Dotted line: Same as in B. In B and C, the vertical axis extends from 0 to 1.

The thin lines of Fig 7A represent the amount of Fisher information obtained for each of the 11 players.

The thick line is the sample average of the thin lines, and the gray area represents the standard error of the sample mean. Individual players exhibit a sequence of maxima and minima, implying that they recall some colors accurately (the maxima) and some others, poorly (the minima).

The local maxima in *J*(*t*) represent colors that are particularly well recalled. Not all local maxima, however, have the same relevance; only those with particularly large numerical value are noteworthy, since $\sqrt{J(t)}$ determines the distance between colors (Eq 8). For each player, therefore, we identified all the local maxima of *J*(*t*) that were at least as large as $\bar{J}+2S_{J}$, where $\bar{J}$ is the average value of *J*(*t*) throughout the interval *t* ∈ (−*π*, *π*], and *S*_*J*_ is the standard deviation. The collection of such maxima obtained for the 11 players is displayed in Fig 7B. In turn, the local minima of *J*(*t*)—or equivalently, the maxima of 1/*J*(*t*)—represent colors that are particularly difficult to recall. Since again, the numerical value of the minimum is relevant, for each player we identified the local minima in *J*(*t*) whose value was such that 1/*J*(*t*) was larger than $\bar{J^{-1}}+2S_{J^{-1}}$. These minima are displayed in Fig 7C. For comparison, panels B and C also display in dotted line the theoretical cumulative distribution expected under the hypothesis of uniform probability density. A Smirnov-Kolmogorov test evaluating the difference between the empirical distributions and the straight line gives a highly significant result, *p* = 0.00007 in the case of maxima (panel B), and *p* = 0.006 in the case of minima (panel C). We therefore conclude that throughout the collection of sampled players, observers tend to recall the colors cyan, green, yellow and red accurately (clusters in panel B), and colors purple and blue poorly (clusters in panel C).

Once *J*(*t*) is known, it is possible to define a new color scale *s* = *s*(*t*) that is recalled with uniform accuracy by a given observer. Mathematically, this means that *J*(*s*) = cnst.

$$\begin{array}{ccc} \text{cnst} & = & J(s) \end{array}$$ (10)

$$\begin{array}{ccc} & = & -\int P\left(r|s\right)\;\frac{\partial^{2}}{\partial s^{2}}{\text{log}}_{2}P\left(r|s\right)\;dr \end{array}$$ (11)

$$\begin{array}{ccc} & = & -{\left(\frac{dt}{\text{ds}}\right)}^{2}\;\int P\left[r|s^{-1}\left(t\right)\right]\;\frac{\partial^{2}}{\partial s^{2}}{\text{log}}_{2}P\left[r|s^{-1}\left(t\right)\right]\;dr \end{array}$$ (12)

$$\begin{array}{ccc} & = & {\left(\frac{dt}{\text{ds}}\right)}^{2}\;J\left(t\right). \end{array}$$ (13)

Hence,

$$\begin{array}{c} ds={\text{cnst}}^{-12}\;\sqrt{J(t)}\;dt, \end{array}$$ (14)

implying that

$$\begin{array}{c} s\left(t\right)=s_{0}+{\text{cnst}}^{-12}\int_{t_{0}}^{t}\sqrt{J(t^{\prime })}\;dt^{\prime }. \end{array}$$ (15)

**Fig 8:**
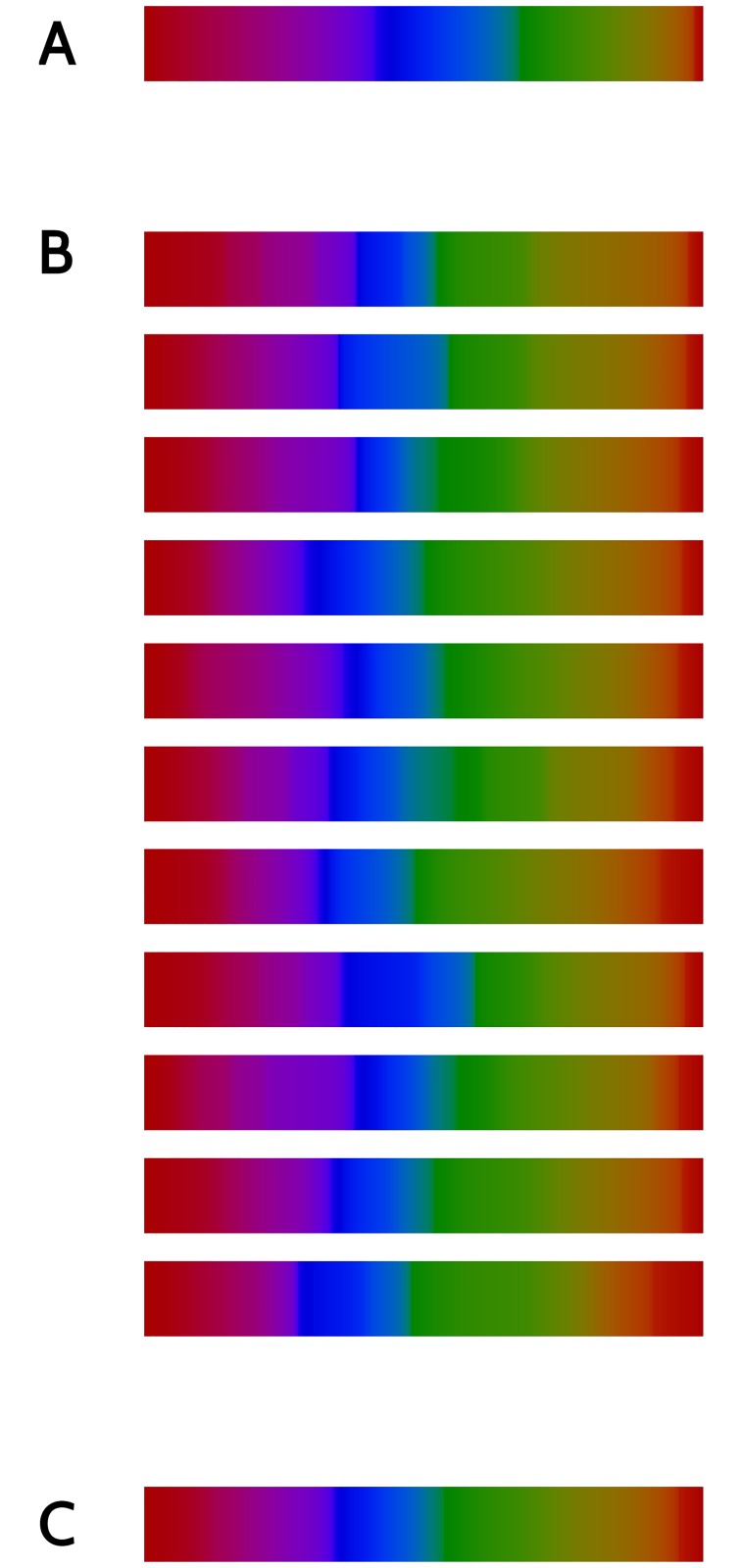
A mnemonically uniform color scale. Comparison between the original color scale *t* used in the game (A) with the new uniform color scales *s* designed for each player (B). C: uniform color scale obtained with the sample average of *J*(*t*).

The proportionality between Eqs 15 and 8 implies that the perceptually uniform variable *s*(*t*) represents the subjective distance between *t* and some reference color *t*_0_. The new notion of subjective distance introduced here represents the cumulative mnemonic discriminability ∑d*s* with which the observer differentiates all pairs of neighboring colors *t*′ and *t*′ + d*t*′ along the way from *t*_0_ to *t*. In Fig 8 we compare the original color scale *t*, with the new uniform scale *s*(*t*) individually tailored for each observer.

In all cases, we see that the violet-blue region is compressed, whereas the green-yellow-red range is expanded. At the bottom, the uniform color scale of the average observer is displayed, and quite evidently, it differs only little from the scales of individual observers.

The amount of Fisher information is calculated with Eq 6, which contains two positive terms. The first term weighs the contribution of the rate of change in *μ*(*t*). Colors for which *μ*(*t*) varies rapidly with *t* allow observers to detect differences between neighboring colors. The second term weighs the rate of change in *σ*^2^(*t*). For all subjects, the first term was always more important than the second. The ratio first/second ranged between 4.1 and 57 for all players (mean 19, SD 14). The rate of change of *σ*^2^(*t*), hence, has a relatively minor effect.

The mean responded color *μ*(*t*) is equal to *t* + Δ(*t*), so its derivative is *μ*′(*t*) = 1 + Δ′(*t*). The relative contribution of the 1 and the Δ′(*t*) terms is also uneven. The ratio of the former to the latter ranged between 1.9 and 62 for the different subjects (mean 16, SD 21). As a consequence, for many subjects, the global behavior of *J*(*t*) was mainly determined by 1/*σ*^2^(*t*). The experimental data *ϵ*(*t*) that were used to fit the continuous function *σ*^2^(*t*) are displayed in Fig 5. The large value of *σ*(*t*) in the violet/blue region, and its drop in the green/yellow/red zone parallels the behavior of *J*(*t*)^−1^.

In Fig 7, the Fisher information *J*(*t*) seems to have roughly the same behavior for all players. This result may seem at odds with the previous finding, that the location of attractors and repulsors varies from player to player. The location of attractors and repulsors, however, is only weakly linked to the behavior of *J*(*t*).

To see the link between the two, we rewrite Eq 6 as

$$\begin{array}{c} J\left(t\right)={\left(\frac{1+\Delta^{\prime }\left(t\right)}{\sigma (t)}\right)}^{2}+\frac{1}{2}{\left(\frac{{\left[\sigma^{2}\left(t\right)\right]}^{\prime }}{\sigma^{2}\left(t\right)}\right)}^{2}. \end{array}$$ (16)

**Fig 9:**
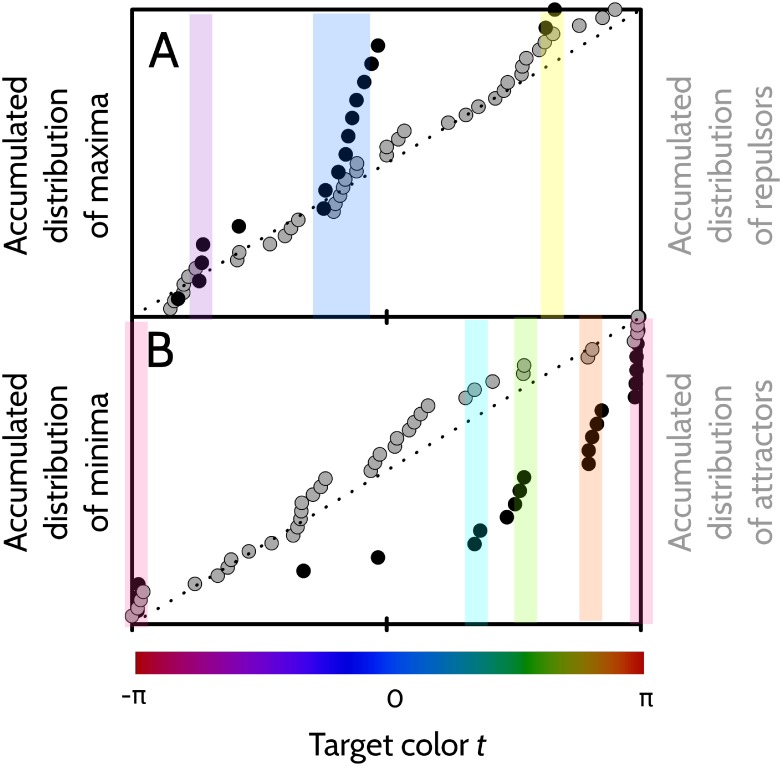
Relation between attractors, repulsors and Fisher information. Comparison of the locations of attractors and repulsors of Fig 6 with that of prominent maxima and minima in *J*(*t*) (Fig 7). A: Empirical cumulative distribution of repulsors (gray) and of prominent maxima of the Fisher information (black). Colored boxes highlight the regions of color space where both distributions appear to increase with slope larger than unity. B: Empirical cumulative distribution of attractors (gray) and of prominent minima of the Fisher information (black). Colored boxes: same as in A. In both panels, the vertical scale ranges between 0 and 1.

Attractors and repulsors are located at colors *t* in which Δ(*t*) = 0, and Δ′(*t*) ≶ 0. From Eq 16, we see that a positive sign of Δ′(*t*) (a repulsor) will tend to increase *J*(*t*), whereas a negative sign (an attractor), to decrease it. Indeed, finer discriminations are expected in regions where the mean response varies with the target color more steeply than the correct response (the error has a positive derivative), and coarser discriminations, where the mean response is shallower. It is therefore natural to expect the Fisher information to drop around attractors, and peak around repulsors. Yet, since the role of Δ′(*t*) is comparatively small when compared with unity, and since *J*(*t*) is more determined by *σ*(*t*) than by Δ(*t*), the mapping between the shape of Δ′(*t*) and of *J*(*t*) cannot be expected to be highly predictable. The loose dependence between the two variables, however, may still be weakly perceived in Fig 9, where the locations of attractors and repulsors are compared to the positions of prominent maxima and minima of *J*(*t*). There seems to be a certain degree of coincidence in the locations where the two sets of data points increase rapidly, marked by the colored bars.

## 3 Discussion

Here we aimed at testing chromatic memory as devoid as possible from the interference with other cognitive abilities. We therefore designed a task where stimuli consisted of large squares of uniform color, lacking spatial structure, texture, and intuitive semantic associations.

In order to work with a circumscribed set of colors, we selected a closed locus in color space. The chosen locus contained hues with the maximal saturation attainable from a computer screen. A set of 32 target colors was chosen from the locus, and the accuracy with which these colors were stored and retrieved from memory was measured. The retrieval error was calculated as the angular distance between the target and retrieved colors. This notion of error naturally depends on the chosen coordinates to designate different colors on the locus. If a nonlinear transformation *t*′ = *φ*(*t*) is used to define a new chromatic scale, the colors that yielded accurate responses in the scale *t* need not yield an accurate response in the scale *t*′. In our study, color coordinates were defined by the physical properties of the light spectrum corresponding to each stimulus. Specifically, two neighboring colors associated with spectra *E*(λ) and *E*(λ) + *δE*(λ) were associated with angles *t* and *t* + *δt*, where d*t* was proportional to *∫*|*δE*(λ)|dλ. All our measures must therefore be interpreted with reference to this objective scale. For example, if for a given target color *t*, an observer produces particularly accurate responses, we may conclude that he or she can accurately represent the difference *δE*(λ) from neighboring colors.

### 3.1 The accuracy of chromatic memory

The first main conclusion is that colors in the yellow-orange region are memorized more accurately than in the violet-blue region. The effect was highly significant throughout the 11 players, and was evident both in the mean error, and its standard deviation. We emphasize that this result is linked to the choice of coordinates reflecting the physical properties of spectra. The same result was observed in [34] and [26].

### 3.2 Attractors and repulsors

The mean retrieval error of the 11 players was significantly different from zero for many target colors. The mean error Δ(*t*) could be satisfactorily fitted with a smooth periodic function. The zeros of the fitted function represented target colors that yielded unbiased responses. Among them we could identify several target colors that were significantly stable to variations in the fitted function, defining attractors and repulsors. Attractors correspond to focal colors, since they tend to concentrate the responses to neighboring colors. Repulsors, in contrast, separate the basins of attraction of two attractors, and therefore, lie at the border between two categories. The location of attractors and repulsors does not depend on the choice of coordinates. The widths of categories, however, defined as the separation of the two repulsors to either side of an attractor, may depend on the choice of coordinates.

Our significance criterion to define attractor and repulsor colors for each player was strict, and still, all players revealed the presence of attractors and repulsors. We therefore conclude that observers make use of at least some degree of categoric strategies. When conversing with the players, we observed that they often made reference to mnemonic strategies based on the association of the target color with some familiar object with a similar hue. Examples such as “the color of my couch”, “the color of my partner’s eyes”, or “the color of my favorite pair of slippers” were heard frequently. We hypothesize that such personal referent objects gave rise to idiosyncratyc focal colors. The absence of clustered attractors and repulsors in the whole collection of sampled players hints to the hypothesis that each player had his or her own private collection of mnemonic referents. Yet, it may still be the case that our failure to confirm a significant degree of clustering may be due to our limited number of subjects.

The presence of attractors and repulsors confirms the findings of Bae et al. [26, 27], who show that the width of response distributions is not constant throughout color space, and that response distributions are determined, at least to a certain extent, by the way observers categorize colors. Our results are also compatible with their model combining categories and particulars in chromatic working memory [27]. The most important difference between our results and theirs is that they seem to obtain more consistency across subjects in the location of attractors.

Consistency across observers can be interpreted in two different ways: As an artifact of the choice of coordinates, or as a property of a universal mnemonic strategy shared by all the sampled players. The first interpretation holds if the width of the response distributions is a direct consequence of the coordinates of color space being too densely (or dispersedly) parsed in that region. If this is the case, some regions of color space should produce broad response distributions for all sampled players, and others, narrow. Moreover, the regions of color space producing broad distributions in the mnemonic task should also produce broad distributions in perceptual tasks, or in color naming tasks, since the breadth is supposed to arise from an inherent property of the coordinates. A nonlinear transformation of the coordinates should exist, producing more uniform response distributions for all players, and all behavioral paradigms. In fact, the pursuit for a nonlinear transformation that homogenizes the variable discrimination ellipses initially measured by MacAdam [2] has lead to the definition of several alternative color spaces, as the UCS diagram CIE 1960, the CIELUV and CIELAB coordinates [46, 47], and the individually tailored space of da Fonseca and Samengo [31].

The second interpretation, instead, is likely to hold if the breadth of the response distributions in a mnemonic task is not congruent with the breadths obtained in perceptual or naming tasks, or in other mnemonic tasks performed in different behavioral conditions. For example, in [18] the responses of a speeded task are compared to those conducted at a comfortable velocity, and the responses of naïve observers are compared to those of trained ones.

Both [27] (testing memory) and [18] (testing discrimination) compare the responses of pairs of colors astride the border defining two linguistic categories, and pairs that fall on the same category. In the present study, we identify focal colors (attractors), and category boundaries (repulsors) with criteria constructed with respect to a physically defined chromatic scale. Hence, our categories are not anchored to the linguistic definition. The subject-to-subject variability that we find rules out a spurious effect purely based on the choice of coordinates, and hints to idiosynchratic categories.

### 3.3 A mnemonically uniform color scale

The amount of Fisher information *J* at a certain color *t* quantifies the ability of a player to store two similar target colors *t* and *t* + d*t* and recall them as distinct. One naïvely expects that such ability is diminished around attractors, and enhanced around repulsors, since the former focus responses, whereas the latter defocuses them. The Fisher information, however, is not entirely determined by the behavior of the mean response *μ*(*t*), actually, in the recorded data, the dependence of the variance *σ*^2^(*t*) on the target color *t* is more relevant. The main trend in *J*(*t*), related to the fact that the information is low in the violet/blue region and high in the green/yellow/orange/red region, is indeed explained by the behavior of *σ*^2^(*t*). Still, Fig 9 suggests that there might be some degree of coincidence between the location of attractors and repulsors on one side, and that of minima and maxima in *J*(*t*), on the other. Since no significant clustering was detected in the location of attractors and repulsors, we simply take this finding as a suggestion, without drawing a definite conclusion from it. An experiment performed with a larger sample may settle the matter.

The amount of Fisher information is not only useful to measure discriminability between neighboring colors, but more importantly, to construct a mnemonically uniform color space individually tailored for each observer. The uniform color space is defined as the one in which the differential d*s* is equal to $\sqrt{J(t)}\;dt$, in the same spirit of previous studies [48, 49]. We have found that even in spite of individual differences in the location of attractors and repulsors, the integrals are quite similar throughout the collection of sampled players, giving rise to a collection of congruent mnemonically uniform spaces (Fig 8). The uniform scale does not depend on the coordinates in which the original experiment was performed. The integration process compensates for the variations in the lengths of the differentials corresponding to different coordinates.

In the new scale, the classical color categories “red”, “purple”, “blue”, “green” and “orange” comprise intervals whose lengths are more uniform than in the original scale. For example, in the upper stripe of Fig 8, the blue sector is clearly longer than the orange one, and this discrepancy becomes less evident in the lower stripe. The mnemonically uniform scale contracted the blue region, in view of its large errors. This result is in agreement with the findings of Bae et al. [27], where the width of response distributions scaled with the width of color categories. A similar effect was found by Witzel and Gegenfurtner [17], when assessing just-noticeable differences in a perceptual discrimination task. The discrimination ability between two colors that belonged to categories (for example green, or red) occupying a large sector of the space (no matter whether DKL, CIELUV or CIELAB) was lower than in narrow sectors. In all these cases, the co-variation between the width of color categories and the width of response distributions hints to a certain arbitrariness in the choice of coordinates in the tested color space. The new mnemonically uniform color scale is designed to compensate for this arbitrariness. Performing future experiments in this compensated scale would be equivalent to the carefully calibrated experiment of Witzel and Gegenfurtner [18], where residual effects of categories are assessed in discrimination experiments in which the perceptual irregularities are evened out.

The integral of *J*(*t*) d*t* can always be performed, so for 1-dimensional spaces, as the one explored here, it is always possible to find a coordinate transformation that defines a new space where the Fisher information is a scalar and constant matrix. Such a coordinate transformation, however, is not guaranteed to exist in experiments testing higher-dimensional color spaces. The Fisher tensor defines not only the metric, but also the curvature of the space. And although the components of the metric depend on the choice of coordinates, the curvature is invariant. The Fisher matrix of a uniform color space is proportional to the identity matrix, and the curvature of such a space necessarily vanishes everywhere. Therefore, only flat spaces (i. e., with zero curvature) may be transformed into a uniform space. Moreover, a vanishing curvature is a sufficient condition for the transformation to be achievable. The new coordinates can be found by integrating the geodesic equation, using the vectors of an orthonormal base starting from a given point as initial conditions [50, 51]. Previous theoretical work performed on chromatic perception (as opposed to chromatic memory) defined a perceptually uniform color space using this procedure, starting from a Fisher matrix that described the physiological properties of cones [31]. Another study, describing the nonlinearities and the adaptation phenomena taking place in the first stages of visual processing, implemented this integration procedure numerically [52]. Both examples were successful, because they operated in flat spaces. The feasibility of the pursuit of uniform coordinates in higher-dimensional color spaces, hence, hinges upon the curvature of the space of remembered colors, which is in turn determined by the way in which the size, shape and orientation of the ellipses describing the variance of the responses vary throughout color space.

## 4 Methods

### 4.1 Calibration of screen colors

The color of each pixel on the screen is determined by coordinates (*R*, *G*, *B*) set by the program, yielding 3-dimensional space. Achromatic stimuli are located along the direction *α* (1, 1, 1), with pure black at the point (0, 0, 0) (that is, *α* = 0), and increasingly lighter shades of gray corresponding to larger *α* values. Saturated colors, instead, are located in the regions of space that are as far from the (1, 1, 1) direction as possible. These regions coincide with the coordinate planes, that is, at *R* = 0 (from blue, to cyan, to green), *G* = 0 (from red, to purple, to blue), and *B* = 0 (from green, to yellow, to red). The *RGB* coordinates used in the game defined a closed locus in color space. The colors on the locus were chosen with the criteria described in Sect. 2.2.

**Fig 10:**
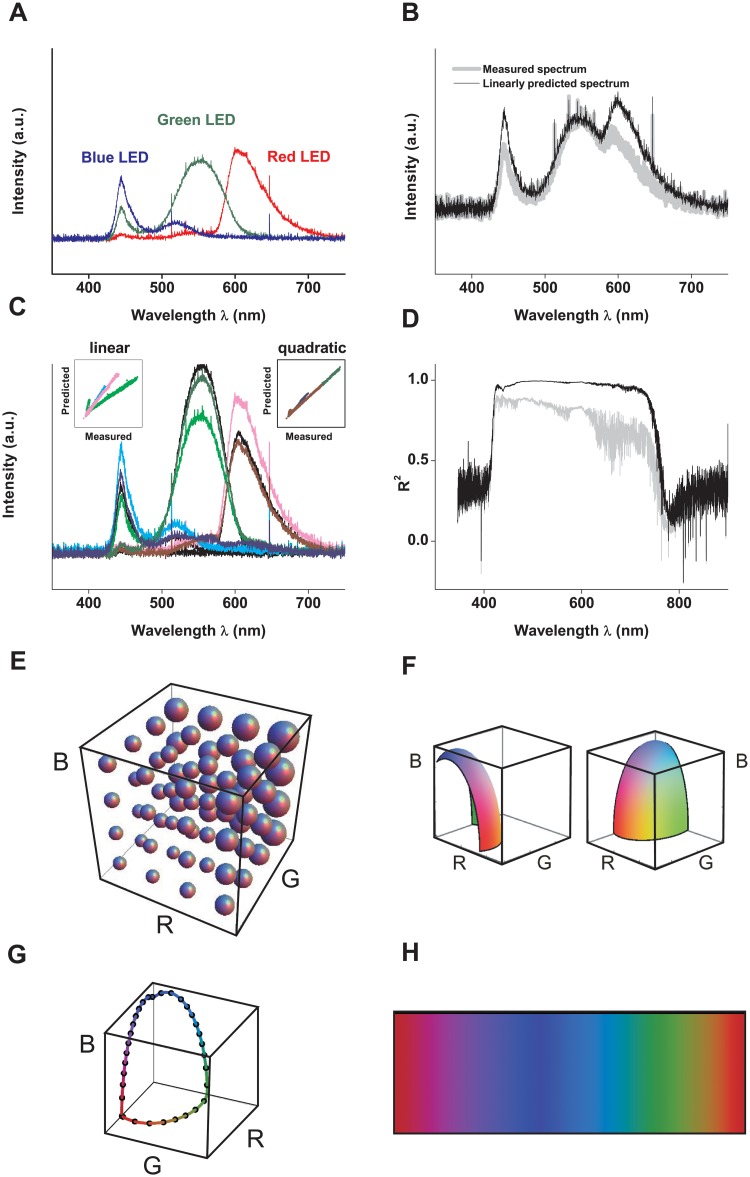
Calibration of the game. A: Spectra of the three LEDs of the computer screen, with coordinates (128, 0, 0) (red), (0, 128, 0) (green) and (0, 0, 128) (blue). B: Measured (gray) and linearly predicted (black) spectra, for a gray screen where *RGB* = (128, 128, 128). C: Black curves: measured spectra for *RGB* = (255, 0, 0), (0, 255, 0) and (0, 0, 255). Light colors (pink, light green and cyan): spectra predicted with the linear model. Dark colors (red, green, blue): predictions for the quadratic model. Inset: comparison between predicted and measured intensities for the two models. D: Square residuals of the two proposed models as a function of wavelength. Black: quadratic model. Gray: linear model. E: Total light intensity as a function of the coordinates R, G and B. Intensity is represented by the volume of each sphere. Each axis ranges between 0 an 255. F: Quadratic fit of a surface with constant intensity. Each axis ranges between 0 and 255. G: Locus of colors used in the game. Continuous colored curve: the 743 colors swept by the scrolling bar. Black points: the 32 target colors. H: Linear parametrization of the colors displayed in G.

Each pixel in the computer screen can be instructed to emit light with controlled *RGB* intensities, by regulating the amount of energy emitted by the three corresponding LEDs. The spectra of the LEDs in the computer screen used in the experiment (*Dell Inspiron N5010*) were recorded with a spectrometer *Ocean Optics USB* controlled with the software *Spectra Suite*. The spectral power densities are displayed in Fig 10A. The coordinates *R*, *G* and *B* could vary between 0 and 255. The mapping between the *RGB* coordinates and the light intensity on the computer screen was non-linear, as shown in Fig 10B. That is, when the screen represented the coordinates (128, 128, 128), the measured spectrum differed from the one predicted by adding the three spectra in Fig 10A. We therefore constructed a quadratic model that allowed us to predict the spectrum generated by the screen as a 2^nd^ order polynomial of the *RGB* coordinates. The quadratic model was significantly more accurate than a linear model (compare Fig 10C and 10D).

The quadratic model provided an analytical expression of the spectrum for each *RGB* trio, with which it became possible to calculate the amount of energy for each point in *RGB* space, as illustrated in Fig 10E. Intensity grows fastest in the direction of *G*, at an intermediate speed in the direction of *R*, and slowest in the direction of *B*. These features are not surprising, since the human visual system is most sensitive to light intensity of intermediate wavelengths [53], and screen technologies are designed to match the visual capacities of human observers.

The quadratic nature of the mapping implied that the iso-intensity colors required by criterion 1 of Sect. 2.2 formed an ellipsoid in the *RGB* space (Fig 10F). The colors of the game belonged to the surface of the maximal ellipsoid that could be fit in the range of *RGB* values emitted by the screen. To compensate for the screen’s intensity dependence on hue, the selected ellipsoid had large *B* values, intermediate *R*, and comparatively small *G* values. To satisfy the saturation condition imposed by criterion 2, the colors of the game were chosen as the locus defining the outer borders of the chosen ellipsoid, that is, the locus lying on the coordinate planes. The step between two consecutive colors was chosen according to criterion 3 defined above, giving rise to the curve in Fig 10G. The linear parametrization of the colors of the scrolling bar are displayed in Fig 10H. Table 1 contains a the CIE xy coordinates or the target colors.

**Table 1:** CIE *xyY* coordinates of the tested target colors.

x	y	Y	x	y	Y
0.640	0.330	8.293	0.582	0.298	8.332
0.502	0.254	8.250	0.427	0.212	8.147
0.362	0.177	8.041	0.310	0.148	7.937
0.268	0.125	7.833	0.233	0.106	7.721
0.207	0.091	7.600	0.188	0.081	7.454
0.174	0.073	7.246	0.162	0.067	6.889
0.151	0.060	5.394	0.151	0.064	6.384
0.154	0.074	7.997	0.160	0.096	9.736
0.167	0.120	10.955	0.175	0.151	12.056
0.195	0.221	12.365	0.201	0.244	13.997
0.219	0.309	14.808	0.242	0.390	15.527
0.268	0.485	16.121	0.294	0.577	16.450
0.308	0.593	18.203	0.342	0.567	19.117
0.390	0.529	18.420	0.443	0.487	17.066
0.503	0.438	15.237	0.566	0.389	13.051
0.616	0.349	10.767	0.638	0.331	8.577

Since the locus of responded colors is closed, target and responded colors are labeled with an angle in (−*π*, *π*]. All averages and standard deviations of colors, hence, are calculated using circular statistics. That is, for a collection of angles *r*_1_, …, *r*_*n*_, the mean value is calculated as

$$\begin{array}{c} \bar{r}=\text{ArcTan}\left(S_{n}/C_{n}\right)\;\;\;\;\text{with}\;S_{n}=\frac{1}{n}\sum_{i=1}^{n}\text{sin}\left(r\right),\;\;\;C_{n}=\frac{1}{n}\sum_{i=1}^{n}\text{cos}\left(r\right). \end{array}$$ (17)

In turn, the standard deviation is calculated as

$$\begin{array}{c} \epsilon =-2\text{log}(\sqrt{S_{n}^{2}+C_{n}^{2}}). \end{array}$$ (18)

### 4.2 Data collection

The game was played by 11 subjects, 5 females, 6 males, with ages between 23 and 43. They had normal or corrected-to-normal visual acuity, and they all had normal color vision, as assessed by the Farnsworth-Munsell 25-hue color vision test [54]. All players were native Spanish speakers, and were fluent in English. They all gave their written informed consent. The experiment was approved by Insituto Balseiro’s ethics committee.

Subjects were not time-constrained to select the responded color, and they often explored the bar at leisure. In all trials, the colors of the bar were displayed with the same ordering, with the middle position of the cursor corresponding to cyan, and the two extremes to pinkish-red. Although in principle this constancy could induce a motor bias, we observed that players, before selecting the responded color, they typically oscillated back and forth around their zone of interest, so that the final choice was approached sometimes from the left and sometimes from the right. Due to the long duration of each game (≈ 15 minutes), some players could not avoid an occasional distraction, which meant that they missed the target color completely. On such occasions, they had no alternative but to respond a randomly selected color. Such responses do not reflect the discriminability of remembered colors, since no color was remembered. To discard such (rare) events, all responses that differed in more than 3 standard deviations from the mean response of that player to each presented color were discarded, and not used in the analysis. The total number of discarded responses was smaller than 0.3% of the total number of responses.

### 4.3 Fitting the mean response error

In Eq 4, Δ(*t*) is defined in terms of 2*m* + 1 parameters s *a*_*j*_ and *b*_*j*_, and *m* determines the maximal frequency of the expansion. The optimal fitting parameters $a_{i}^{\text{opt}}$ and $b_{i}^{\text{opt}}$ were obtained by minimizing the sum of the squared differences

$$\begin{array}{c} \chi^{2}=\sum_{j=1}^{32}{\left[\frac{\bar{r}\left(t_{j}\right)-\mu \left(t_{j}\right)}{\epsilon \left(t\right)/\sqrt{n_{j}}}\right]}^{2}, \end{array}$$ (19)

where *n*_*j*_ is the number of times that the player responded to the color *t*_*j*_. Under the assumption that fluctuations are Gaussian, minimizing the value of *χ*^2^ is equivalent to a maximum-likelihood estimation. The optimal coefficients were calculated for increasing *m* values, starting from *m* = 2. The best model is the one with the optimal *m* value, and for that *m*, the coefficients obtained from the maximum likelihood estimation. To select the optimal *m* value, we considered the fact that as *m* grew larger, the fitting accuracy of Eq 4 improved, but at the cost of increasing the risk of overfitting. To evaluate the trade-off between these two factors, for each *m* value we constructed the null hypothesis that the data were generated from a normal distribution of mean *μ*(*t*) = *t* + Δ(*t*) and variance *ϵ*^2^(*t*), where the shape of Δ(*t*) is defined by the fitted parameters *a*_*i*_ and *b*_*i*_ that were optimal for the chosen *m* value. Under the null hypothesis, the probability of obtaining a *χ*^2^ value at least as large as the one as the numerical value obtained from Eq 19 is

$$\begin{array}{c} p_{\text{value}}=\int_{\chi^{2}}^{+\infty }P_{\text{df}}\left(z\right)\;dz, \end{array}$$ (20)

where *P*_df_(*z*) is the *χ*^2^ distribution with dg = 32 − (2*m* + 1) degrees of freedom. Hence, each fitted coefficient in the expansion of Eq 4 subtracts one degree of freedom of the distribution *P*_df_(*z*), thereby properly weighing both the improvement and the drawback of adding new parameters. The null hypothesis is accepted, unless the *P*_value_ is too small to make the null hypothesis plausible. We defined the optimal *m* as the one yielding the smallest local maximum in the curve *P*_value_(*m*) for which *χ*^2^ ≤ 32. The optimal *m*-values ranged from 4 to 12 (mean 8.2, SD 2.7).

**Fig 11:**
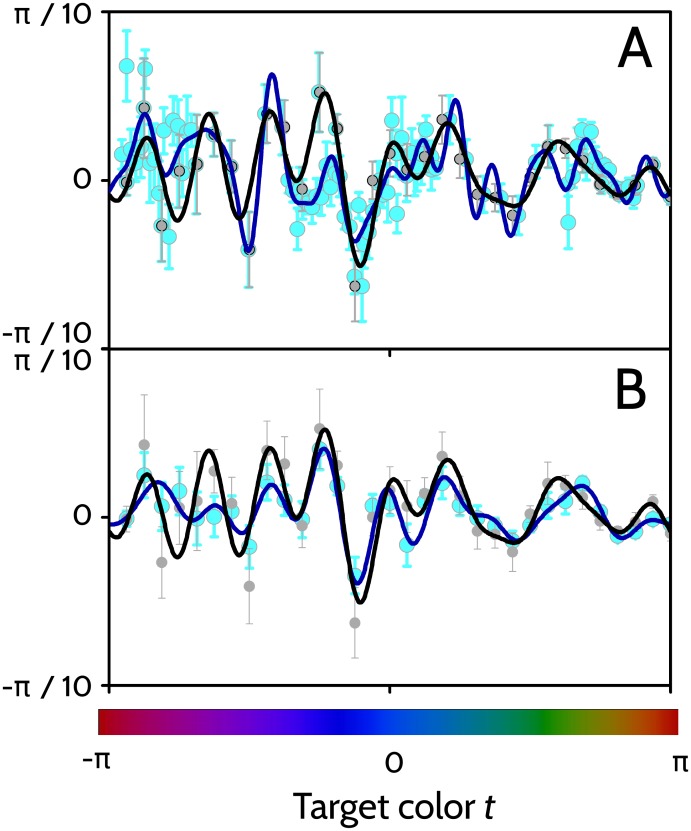
Fitted errors Δ(*t*) in two control experiments. A: Fitted error obtained for an observer that played the normal 32-color game (gray data points, black fitted curve), and a modified 96-color game (cyan data points, blue fitted curve). Both games were played 10 times. B: Fitted error obtained for an observer that played the normal 32-color game 10 times (gray data points, black fitted curve), and 26 times (cyan data points, blue fitted curve).

To verify whether the recorded data allowed us to make reliable estimates of the fitted functions Δ(*t*), two control experiments were performed. The first control (Fig 11A) determined whether the 32 sampled colors sufficed to obtain a continuous curve Δ(*t*), without missing some relevant structure in the intermediate, non-sampled colors.

One of the observers played the normal 32-color game, and also a modified version of the game that tested 96 target colors, with fine graining in three regions of the chromatic locus: a magenta zone, a blue zone and a yellow zone. The fitted error functions Δ(*t*) obtained with coarse and fine sampling differed only little from one another, with very few discrepancies in the green-yellow-red zone, and some more in the magenta-blue region. The difference between the curves was in all cases of the same order of magnitude as the standard error of the mean responded color, obtained from the trial-to-trial fluctuations. The second control tested whether playing the game 10 times was sufficient to estimate Δ(*t*). One of the observers played the game 26 times, and the derived curve Δ(*t*) was compared with the one obtained by using only the first 10 trials (Fig 11B). The derived fits were similar, and their difference was always of the same order of magnitude as the expected error of the mean response calculated with 10 trials.

### 4.4 Significance of attractors and repulsors

An attractor (repulsor) is defined as a target color *t* for which Δ(*t*) = 0, and dΔ(*t*)/d*t* < 0 (dΔ(*t*)/d*t* > 0). However, the fitted error Δ(*t*) may change sign due to the fact that the amount of recorded data is limited. Due to chance alone, fluctuations in a certain region of target colors may well have a different sign from fluctuations in some other region. Since we associate a cognitive function to attractor and repulsor colors, it is important that a given target color *t* only be considered an attractor or a repulsor if we are confident that the conditions Δ(*t*) = 0 and dΔ(*t*)/d*t* ≶ 0 hold beyond stochastic fluctuations. To assess whether such is the case, we constructed a significance criterion for identifying attractor and repulsor colors, by evaluating the degree of certainty in the fit Δ(*t*), and the degree up to which the condition dΔ(*t*)/d*t* ≶ 0 holds not only for the optimal fit, but also, for a whole family of fitting functions that, though not optimal, are still in some sense *near* the optimal fit.

Maximum likelihood estimation produces the optimal parameters $a_{i}^{\text{opt}}$ and $b_{i}^{opt}$, as well as their expected estimation errors. Since the parameters form a (2*m* + 1)-dimensional vector, the errors are captured by a (2*m* + 1) × (2*m* + 1) covariance matrix *C*. The diagonal terms *C*_*ii*_ are the expected square errors of the fitted parameters, and the non-diagonal terms, their mean-subtracted correlations. We can therefore assume that the vector of coefficients **q** = (*b*_0_, *a*_1_, *b*_1_, …, *a*_*m*_, *b*_*m*_) is governed by a multivariate Gaussian distribution of mean $q_{0}=\left(b_{0}^{\text{opt}},a_{1}^{\text{opt}},b_{1}^{\text{opt}},\ldots ,a_{m}^{\text{opt}},b_{m}^{\text{opt}}\right)$ and covariance matrix *C*. In other words,

$$\begin{array}{c} \text{Prob}\left(q\right)=\frac{\text{exp}[-\left(q-q_{0}\right)C^{-1}{\left(q-q_{0}\right)}^{t}/2]}{{\left(2\pi \right)}^{(2m+1)/2}\sqrt{\det C}}, \end{array}$$ (21)

where the supra-script *t* indicates vector transposition. Assessing whether the condition dΔ(*t*)/d*t* ≶ 0 holds with a certain degree of significance amounts to evaluating the fraction of models (weighted with the distribution of Eq 21) for which the condition is verified. A highly significant attractor is one for which a large fraction of models fulfill the condition. Here we accepted a target color *t* as a significant attractor only if the set of suboptimal fits Δ(*t*) that captured 95% of the probability of the distribution of Eq 21 still verified the condition dΔ(*t*)/d*t* ≶ 0. We also verified that with this criterion, simulated responses obtained from a fictional player with flat Δ(*t*), and who played the game the same number of times as the real players, never gave rise to significant attractors or repulsors.

## Supporting information

S1 File Experimental data. Raw list of responses, trial by trial, of the 11 sampled players to each of the 32 target colors, in *xlsx* format.
(XLSX)
